# Prevalence of *SERPINA1* mutations in a bronchiectasis cohort: implications of extended screening for alpha-1 antitrypsin deficiency

**DOI:** 10.36416/1806-3756/e20250181

**Published:** 2025-11-14

**Authors:** Caroline Souza Sokoloski, Mariane Gonçalves Martynychen Canan, Cleverson Alex Leitão, Karin Mueller Storrer

**Affiliations:** 1. Serviço de Pneumologia, Complexo Hospital de Clínicas, Universidade Federal do Paraná, Curitiba (PR) Brasil.; 2. Serviço de Radiologia, Complexo Hospital de Clínicas, Universidade Federal do Paraná, Curitiba (PR) Brasil.

**Keywords:** Alpha 1-antitrypsin deficiency, Bronchiectasis, Genotype, Mutation, Alleles

## Abstract

**Objective:**

To evaluate the prevalence of alpha-1 antitrypsin (AAT) variants through SERPINA1 genotyping in patients with non-cystic fibrosis bronchiectasis, and assess their clinical, functional and radiological characteristics. AAT deficiency is underdiagnosed, and an etiology to be considered when evaluating bronchiectasis.

**Methods:**

A cross-sectional study was conducted at an outpatient clinic focused on bronchiectasis in a tertiary hospital. Data from patients followed between 2005 and 2023 were collected. Genotyping for AAT was performed. Demographic, clinical, pulmonary function tests, serum AAT levels and chest CT data were analyzed.

**Results:**

A total of 136 patients were included, predominantly female (72.1%), with a median age of 56.6 years. The prevalence of SERPINA1 gene mutations was 25.7% (n=35). Among the detected variant genotypes were Pi*MS (15.4%), Pi*MZ (5,1%), Pi*SS (1,5%), Pi*ZZ (1,5%), Pi*MI (0,7%), Pi*SZ (0,7%) and Pi*ZMMalton (0,7%). When comparing patients with and without SERPINA1 mutations, significant differences were observed in AAT serum levels, emphysema type (panlobular) and distribution (diffuse and lower-lobe predominant). No other clinical, microbiological, functional or radiological differences were found, including emphysema presence or absence. Notably, 16 (45.7%) of individuals carrying SERPINA1 mutations exhibited normal serum AAT levels.

**Conclusions:**

AAT variants are not uncommon among patients with bronchiectasis. Presence of panlobular, diffuse or lower-lobe predominant emphysema should prompt AATD diagnostic consideration. However, the absence of emphysema does not exclude the diagnosis. Moreover, SERPINA1 variants may occur along with normal AAT serum levels. Clinicians should consider genotyping in patients with normal AAT levels, particularly when bronchiectasis remains unexplained.

## INTRODUCTION

Alpha-1 antitrypsin deficiency (AATD) is a still underdiagnosed autosomal codominant disorder caused by mutations in the *SERPINA1* gene, resulting in reduced serum levels of alpha-1 antitrypsin (AAT) and increased susceptibility to chronic pulmonary and liver diseases.^1^ AAT is the main circulating serine protease inhibitor, and its deficiency promotes a protease-antiprotease imbalance, favoring lung tissue damage to occur.^2^ Although AATD is typically associated with emphysema, recent studies have also linked the condition to other lung diseases, such as asthma and non-cystic fibrosis bronchiectasis.^3^ ^-^ ^5^

Bronchiectasis is a chronic suppurative lung disorder, caused by a heterogenic group of diseases, characterized by permanent bronchial dilation, typically presenting with chronic cough, sputum production, dyspnea and recurrent respiratory infections. Identifying the underlying etiology is critical to guide management and prognosis.^6^ Despite thorough evaluation, 24-40% of bronchiectasis cases remain undetermined worldwide.^7^ ^,^ ^8^ Therefore, AATD should be systematically considered as a possible cause of bronchiectasis.^3^ ^,^ ^6^

Although the prevalence of AATD is unknown in most countries and varies across populations, it is most frequently observed in individuals of European ancestry,^3^ with estimates of up to 1 in 5,000 people in Europe.^9^ In Brazil, epidemiological data of AATD in general population are lacking.^10^ A cross-sectional study involving 926 patients with COPD from five different Brazilian regions found an overall prevalence of 2.8% for AATD.^11^ However, to our knowledge, there are no published data specifically addressing the prevalence of AAT variants among patients with bronchiectasis in Brazil.

This study aims to evaluate the prevalence of AAT variants in a population of patients with bronchiectasis using *SERPINA1* gene mutation testing, and to assess their clinical, functional and radiological characteristics compared to those without mutations.

## METHODS

This was a cross-sectional study, performed at the non-cystic fibrosis bronchiectasis referral center of the Federal University of Paraná, southern Brazil. Between 2005 and 2023, 239 patients were followed at the outpatient clinic. Data were evaluated and collected from July 2022 to December 2023. The present study was approved by the Committee for Ethics in Research on Human Beings under the opinion 5.464.008, and written informed consent was obtained from all participants.

Patients aged 18 years or older with bronchiectasis diagnosed by clinical and tomographic criteria, who had undergone genotyping for AAT variants were included. Patients with insufficient data or a diagnosis of cystic fibrosis were excluded.

Diagnosis and etiology of bronchiectasis were defined according to specific diagnostic criteria.^6^ ^,^ ^12^ For this study, etiology was grouped as follows: post-tuberculosis, post-infectious (other), common variable immunodeficiency, immunoglobulin A deficiency, related to HIV virus infection, other immunodeficiency, related to auto-immune diseases (collagen and inflammatory bowel diseases), primary ciliary dyskinesia, AATD-related, undefined (or idiopathic), and others (involving the diagnosis: Williams-Campbell syndrome, Scimitar syndrome, allergic bronchopulmonary aspergillosis, chronic aspiration).

- Clinical, laboratory, and imaging data were retrieved from electronic medical records and hospital information systems and compiled for analysis. Data related to the following were collected:
- Clinical variables: age, sex, race, smoking status, BMI, long-term oxygen therapy, number of exacerbations and hospitalizations in the previous 12 months of follow-up, comorbidities (asthma and prior tuberculosis), and E-FACED bronchiectasis severity score (calculated based on occurrence of severe exacerbation in the previous year, FEV_1_ % predicted, age, chronic infection by *Pseudomonas aeruginosa*, radiological extent, and dyspnea by modified Medical Research Council scale).
- Laboratory variables: serum AAT level, *SERPINA1* genotype, sputum culture microbiology.
- Pulmonary function: FEV_1_, FVC, FEV_1_/FVC ratio, DL_CO_ (absolute and % predicted) and bronchodilator response were evaluated. FEV_1_ was considered very severely reduced when <30% predicted; severely reduced when 30-49% predicted; moderately reduced when 50-79% predicted and mildly reduced when ≥80% predicted.
- CT scan variables: images were acquired in a multidetector scanner (Aquilion 64; Toshiba Medical Systems, Tokyo, Japan), with a high-resolution protocol (1-mm slice thickness and 0.5-mm increments), and assessed by a chest radiologist. Evaluated parameters included presence, type and distribution of emphysema and bronchiectasis, number of affected lobes, bronchial wall thickening, mucus plugging, tree-in-bud opacities, and prior surgical resections.

### AAT measurement and genotyping

Serum AAT levels were measured by turbidimetry in peripheral venous blood samples. The reference range for this method was 90-200 mg/dL. By protocol, measurements were performed during clinically stable periods, as AAT is an acute-phase reactant and may be elevated during inflammatory or infectious exacerbations.^3^ ^,^ ^10^

Serum AAT concentrations were stratified into three categories according to the AATD diagnostic algorithm^3^ ^,^ ^10^ and the internationally recognized threshold for severe deficiency^13^: ≥116 mg/dL (normal), 57-115 mg/dL (intermediate levels), and <57 mg/dL (severely reduced levels).

Genotyping of the *SERPINA1* gene was performed, regardless of AAT serum levels. It was used a buccal swab collection kit (ORAcollect OCR-100; DNA Genotek, Inc, Ottawa, Canada), preserved in bacteriostatic stabilizing solution. Samples were shipped at room temperature and analyzed by Progenika Biopharma S.A. (a Grifols company, Derio, Spain). DNA was extracted and amplified by PCR, followed by hybridization with allele-specific probes targeting 14 common variants in exons II, III, and V, using Luminex xMAP technology (Luminex Corp., Austin, TX, USA).

Genetic diagnosis of AAT variants was confirmed when a mutation was detected in at least one allele.^3^ ^,^ ^10^ AATD was considered a definite etiology of bronchiectasis when severe genotypes were found, such as Pi*ZZ, Pi*SZ and Pi*ZM(Malton).^14^

### Statistical analysis

Exploratory analysis of quantitative variables was performed by calculating means and standard deviations (mean ± SD) or medians and interquartile ranges (IQR), according to data distribution. Categorical variables were expressed as absolute and relative frequencies. Association analysis between nominal, ordinal, or discrete variables were evaluated using the Chi-square test or Fisher’s exact test, when appropriate. For contingency tables with more than two categories and low expected frequencies, the Fisher-Freeman-Halton exact test was used to ensure statistical validity. For continuous outcome variables, normally distributed data were analyzed using one-way ANOVA, while the Kruskal-Wallis and Mann-Whitney U tests were used for non-normally distributed variables. All statistical analyses were performed with the IBM SPSS Statistics software package, version 29.0 (IBM Corporation, Armonk, NY, USA). A two-tailed p-value < 0.05 was considered statistically significant.

## RESULTS

**Figure 1:**
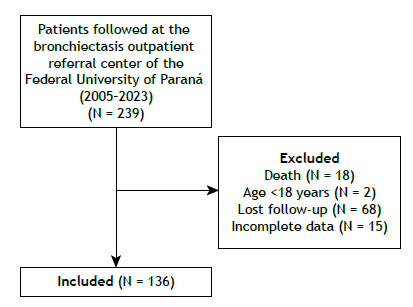
Study flow chart.

Of 239 patients with non-cystic fibrosis bronchiectasis followed at the outpatient referral center between 2005 and 2023, 136 met the eligibility criteria and were included in the study (Figure 1).

Study population characteristics can be seen in Table 1. The majority were female (72.1%) and White (86.8%). Ages ranged from 18 to 85 years, with a median of 59.5 years (IQR: 45.0-67.8). Only 10.3% were underweighted. Most patients were never smokers (71.3%), and from those who were current or former smokers (28.6%), median smoking history was 20 pack-years (IQR: 7-50). 

**Table 1:** Clinical, functional, and radiological characteristics of the study population.

Characteristic(s)	(N = 136*)
Clinical-epidemiological	
Female sex, n (%)	98 (72.1)
Age (years), median (IQR)	59.5 (45-67.75)
White race, n (%)	118 (86.8)
Smoking status, n (%) Smoker Former smoker Never smoker	4 (2.9) 35 (25.7) 97 (71.3)
Smoking history (pack-years), median (IQR)	20 (7-50)
BMI (kg/m^2^), median (IQR) Underweight, n (%) Normal weight, n (%) Overweight, n (%) Obese, n (%)	24.5 (21.92-28) 14 (10.3) 57 (41.9) 45 (33.1) 20 (14.7)
Long-term oxygen therapy, n (%)	15 (11)
E-FACED-based bronchiectasis severity Mild, n (%) Moderate, n (%) Severe, n (%)	89 (65.4) 39 (28.7) 8 (5.8)
Agent of chronic bronchial infection, n (%) None detected *Staphylococcus aureus* *Pseudomonas aeruginosa* *Haemophilus influenzae* Other, n (%) No data, n (%)	78 (57.4) 7 (5.1) 32 (23.5) 3 (2.2) 1 (0.7) 15 (11)
Pulmonary exacerbation in the last year, n (%) 0 1 2 ≥ 3	52 (38.2) 45 (33.1) 24 (17.6) 15 (11)
Hospitalization for severe exacerbation, n (%)	23 (16.9)
Asthma (comorbidity), n (%)	50 (36.8)
Prior tuberculosis, n (%)	38 (27.9)
Alpha-1 antitrypsin serum level ≤ 116 mg/dL, n (%)	28 (20.6)
Lung function^†^	
Obstructive ventilatory defect, n (%) FEV_1_ % predicted, median (IQR) FVC % predicted, median (IQR) FEV_1_ % predicted - stratified, n (%) < 30% 30-49% 50-79% ≥ 80% DL_CO_ % predicted, median (IQR)	105 (78.3) 54.8 (36.7-76.0) 70.6 (56.2-88.1) 20 (14.9) 41 (30.6) 48 (35.8) 25 (18.6) 93.1 (73.9-114.2)
Radiological^‡^	
Emphysema, n (%)	44 (33.3)
Predominant distribution of emphysema, n (%) Upper lobes Lower lobes Diffuse	29 (21.9) 11 (8.3) 4 (3.0)
Predominant type of emphysema, n (%) Paraseptal Centrilobular Panlobular	10 (7.5) 31 (23.4) 3 (2.3)
Predominant distribution of bronchiectasis, n (%) Upper lobes Lower lobes Diffuse	32 (24.2) 60 (45.4) 40 (30.3)
Predominant type of bronchiectasis, n (%) Cylindrical Varicose Cystic	56 (42.4) 50 (37.8) 26 (19.7)
Number of lobes affected by bronchiectasis, n (%) 1 2 3 4 5	7 (5.3) 22 (16.6) 19 (14.4) 20 (15.1) 64 (48.5)
Other, n (%) Bronchial wall thickening Mucus plugging Tree-in-bud opacities Previous pulmonary resection	90 (68.2) 93 (70.4) 87 (65.9) 9 (6.8)

E-FACED: Exacerbation frequency, FEV_1_, Age, Colonization, Extension, and Dyspnea (bronchiectasis severity score). *Varies for some characteristics, as noted below. ^†^Data available for only 134 patients for all characteristics except DL_CO_, for which data were available for only 57 patients. ^‡^Data available for only 132 patients.

Chronic hypoxemic respiratory failure requiring long-term oxygen therapy was present in 15 patients (11%). In terms of clinical outcomes, 28.6% had two or more exacerbations in the last year requiring antibiotic treatment, and 16.9% required hospitalization due to severe exacerbation. According to the E-FACED severity score, 34.5% had severe or moderate disease. 

Microbiological analysis of spontaneous sputum cultures showed that 57.4% of patients did not have chronic bronchial infection. Among those with infection, *Pseudomonas aeruginosa* was the most prevalent pathogen (23.5%).

Pulmonary function tests revealed obstructive ventilatory disorder in 78.3% of patients. Most patients had moderate (35.8%) and severe (30.6%) reduction in forced expiratory volume in one second (FEV_1_).

Considering chest CT, emphysema was identified in 33.3% of patients. When present, it predominantly involved the upper lobes, and centrilobular emphysema was the most frequent pattern. Bronchiectasis was predominantly located in the lower lobes (45.4%) and most frequently cylindrical (42.4%). Extensive lobar involvement (all lobes) was seen in 48.5% of cases. Bronchial wall thickening (68.2%), mucus plugging (70.4%), and centrilobular tree-in-bud opacities (65.9%) were also common. 

Regarding etiology (Table 2), post-tuberculosis bronchiectasis was the most common identified cause (21.3%), followed by post-infectious bronchiectasis due to other pathogens (16.9%). Only 2.9% were attributed to AATD.^15^ The etiology remained undefined in 40.4% of cases, despite extensive investigation.

**Table 2:** Etiology of non-cystic fibrosis bronchiectasis.

Etiology	(N = 136)
Undefined/idiopathic, n (%) Post-tuberculosis, n (%) Post-infectious (other), n (%) Common variable immunodeficiency, n (%) Alpha-1 antitrypsin deficiency*, n (%) Related to auto-immune diseases, n (%) Immunoglobulin A deficiency, n (%) Other immunodeficiency^†^, n (%) Primary ciliary dyskinesia, n (%) Related to HIV infection, n (%) Other^‡^, n (%)	55 (40.4) 29 (21.3) 23 (16.9) 5 (3.7) 4 (2.9) 3 (2.2) 2 (1.5) 3 (2.2) 2 (1.5) 2 (1.5) 8 (5.9)

*Considering only the Pi*ZZ, Pi*ZMMalton, and Pi*SZ genotypes. ^†^Anti-pneumococcal polysaccharide antibody deficiency or immunodeficiency secondary to neoplasm. ^‡^Williams-Campbell syndrome, Scimitar syndrome, allergic bronchopulmonary aspergillosis, or chronic aspiration.

### AAT levels and genotyping

Twenty-eight individuals with bronchiectasis (20.6%) had low serum levels of AAT (≤116mg/dL), including 2.2% with severe deficiency (<57mg/dL) and 18.4% with intermediate concentrations (57-115mg/dL). Furthermore, 35 (25.7%) had at least one *SERPINA1* mutation identified (Table 3).

**Table 3:** Alpha-1 antitrypsin levels and genotyping in a sample of patients with non-cystic fibrosis bronchiectasis.

Genotype	Serum alpha-1 antitrypsin level*			
	Severe	Intermediate	Normal	Total
	< 57 mg/dL	57-115 mg/dL	≥ 116 mg/dL	
	(n = 3)	(n = 25)	(n = 108)	
	n (%)	n (%)	n (%)	n (%)
Pi*MM^†^ AATD variants	0 (0) 3 (2.2)	9 (6.6) 16 (11.7)	92 (67.7) 16 (11.7)	101 (74.3) 35 (25.7)
Pi*MS Pi*MZ Pi*MI Pi*SS Pi*SZ Pi*ZMMalton Pi*ZZ	0 (0) 0 (0) 0 (0) 0 (0) 0 (0) 1 (0.7) 2 (1.4)	7 (5.2) 7 (5.2) 0 (0) 1 (0.7) 1 (0.7) 0 (0) 0 (0)	14 (10.3) 0 (0) 1 (0.7) 1 (0.7) 0 (0) 0 (0) 0 (0)	21 (15.5) 7 (5.2) 1 (0.7) 2 (1.4) 1 (0.7) 1 (0.7) 2 (1.4)

AATD: alpha-1 antitrypsin deficiency. *Determined by turbidimetry. ^†^Healthy genotype.

Presence of *SERPINA1* mutations was found in 100% of patients with severely reduced AAT levels. However, pathogenic variants were also identified in individuals with serum levels ≥57 mg/dL, including some above normal threshold.^3^ ^,^ ^10^

### Comparison between patients with and without SERPINA1 gene mutations

When the cases were stratified by the presence or absence of *SERPINA1* gene mutations (Table 4), significant differences were found for AAT serum levels, emphysema distribution and emphysema type. The median serum AAT level was 107 mg/dL (IQR: 91-130) among mutation carriers, compared to 146 mg/dL (IQR: 131-171) in non-carriers (p < 0.001). Although the overall frequency of emphysema was similar between groups, among patients with AAT mutations, emphysema was more frequently located in lower lobes (57.1%) or diffusely distributed (20%). Panlobular emphysema was observed exclusively in patients with *SERPINA1* mutations (3 cases, 8.6%).

**Table 4:** Comparative analysis between individuals with and without *SERPINA1* gene mutations in a sample of patients with non-cystic fibrosis bronchiectasis.^a^

Characteristic(s)	SERPINA1 mutation		p-value
	No	Yes	
	(n = 101)	(n = 35)	
Clinical-demographic			
Age (years)	60 (48-66)	59 (40-70)	0.823*
Female sex, n (%)	72 (71.3)	26 (74.3)	0.829^†^
BMI (kg/m^2^)	25 (22.3-27.8)	24 (21.6-28.6)	0.615*
Smoking status, n (%) Smoker Former smoker Never smoker	4 (3.9) 29 (28.7) 68 (67.3)	0 (0.0) 6 (17.1) 29 (82.8)	0.193^‡^
Smoking history (pack-years)	15 (7-40)	30 (12-50)	0.412*
E-FACED score	3 (1-4)	2 (1-4)	0.456*
Pulmonary exacerbation(s) in the last year of follow-up	1 (0-2)	1 (0-2)	0.971*
Hospitalization for severe exacerbation in the last year of follow-up	0 (0-0)	0 (0-0)	0.90*
Chronic bronchial infection by *P. aeruginosa*, n (%)	26 (25.7)	6 (17.1)	0.361^†^
Long-term oxygen use, n (%)	13 (12.9)	2 (5.7)	0.353^†^
Prior tuberculosis, n (%)	30 (29.7)	8 (22.9)	0.516^†^
Serum AAT level (mg/dL)	146 (131-171)	107 (91-130)	< 0.001*
Pulmonary function			
FEV_1_/FVC FEV_1_ (% predicted) DL_CO_ (% predicted)	0.63 (0.52-0.73) 49 (36-73) 84.0 (71.9-112.7)	0.63 (0.54-0.75) 57 (46-78) 99.6 (85.3-117.6)	0.632* 0.205* 0.095*
Chest CT			
Number of lobes affected by bronchiectasis	4 (3-5)	5 (2-5)	0.698*
Predominant type of bronchiectasis, n (%) Cylindrical Varicose Cystic	39 (40.2) 38 (39.2) 20 (20.6)	17 (48.6) 12 (34.2) 6 (17.1)	0.689^§^
Predominant distribution of bronchiectasis, n (%) Upper lobes Lower lobes Diffuse	24 (24.7) 40 (41.2) 33 (34)	8 (22.9) 20 (57.1) 7 (20.0)	0.208^§^
Emphysema, n (%)	34 (34.7)	10 (28.6)	0.539^†^
Predominant distribution of emphysema, n (%) Upper lobes Lower lobes Diffuse	26 (76.5) 6 (17.6) 2 (5.9)	3 (30.0) 5 (50.0) 2 (20.0)	0.014^‡^
Predominant type of emphysema, n (%) Paraseptal Centrilobular Panlobular	8 (23.5) 26 (76.5) 0 (0.0)	2 (20.0) 5 (50.0) 3 (30.0)	0.014^‡^
Other, n (%)			
Bronchial wall thickening	70 (71.4)	20 (57.1)	0.143^†^
Mucus plugging	72 (73.5)	21 (60.0)	0.197^†^
Tree-in-bud opacities	66 (67.3)	21 (60.0)	0.535^†^
Previous pulmonary resection	6 (6.1)	3 (8.6)	0.696^†^

AAT: alpha-1 antitrypsin; and E-FACED: Exacerbation frequency, FEV_1_, Age, Colonization, Extension, and Dyspnea (bronchiectasis severity score). ^a^Results expressed as median (IQR), except where otherwise indicated. *Mann-Whitney test. ^†^Fisher’s Exact test. ^‡^Fisher-Freeman-Halton exact test. ^§^Chi-square test.

No statistically significant differences were observed in other demographic, clinical, microbiological, functional, or radiological features.

## DISCUSSION

To our knowledge, this is the first study to report the prevalence of AAT variants in a population of patients with non-cystic fibrosis bronchiectasis in Brazil, in which *SERPINA1* gene mutations were identified in 25.7% of the individuals. It is important to highlight that AATD is a genetically determined condition more commonly observed in Caucasian populations. Given Brazil’s racial admixture and continental dimensions, along with historical differences in regional immigration patterns, it is plausible that the prevalence of AAT variants may vary across regions of the country.

In Brazil, the prevalence of AAT variants was previously assessed only in a cohort of 926 individuals with COPD from five different regions.^11^ In that study, genetic analysis was performed only in the individuals with reduced serum AAT levels identified during initial screening. In contrast, the present study conducted genotyping in the entire sample of patients with non-cystic fibrosis bronchiectasis, which may partially explain the higher prevalence of *SERPINA1* mutations observed (25.7% vs. 2.8%). 

When compared to studies focusing on bronchiectasis populations, our findings also revealed a higher frequency of *SERPINA1* mutations. In Italy, a study that investigated the prevalence of AAT variants by genotyping in a population with bronchiectasis found a frequency of 7.7%.^15^ In the United Kingdom, AATD prevalence among 1,600 patients with bronchiectasis was 0.5%, through initial evaluation of serum levels, followed by phenotyping when levels were low.^16^ In France, AATD diagnosis based directly on phenotyping method in a population of 202 patients with bronchiectasis showed a frequency of 18.81%.^17^

When comparing the two groups-those with and without *SERPINA1* mutations-significant differences were found only in serum AAT levels, predominant pattern of emphysema distribution (lower lobes or diffuse), and emphysema type (panlobular). These findings are consistent with previous reports suggesting that AATD should be suspected in patients with bronchiectasis who exhibit low serum AAT levels and imaging features of basal or panlobular emphysema.^18^ However, it is noteworthy that emphysema was present in only 26.8% of mutation carriers in this sample, and only 30% of these cases had panlobular emphysema. Both cases carrying Pi*ZZ genotype did not have emphysema. Therefore, the absence of emphysema-especially of the panlobular or basal type-should not preclude investigation for AATD. This is further supported by data from the European Alpha-1 Research Collaboration (EARCO) international registry, in which 9.1% of Pi*ZZ carriers presented with bronchiectasis in the absence of radiological emphysema.^4^

Although serum AAT levels were lower in the mutation group, normal values did not exclude the presence of AAT variants. In fact, 16 (45.7%) of the 35 patients with *SERPINA1* gene mutations had serum concentrations above the normal threshold.^3^ Of note, 14 of these 16 patients carried the Pi*MS genotype. The remaining two patients had the following genotypes: Pi*MI and Pi*SS. Although the Pi*MS genotype alone is not historically considered a significant risk factor for the development of pulmonary disease,^14^ ^,^ ^19^ and therefore cannot be considered a definitive etiological factor for bronchiectasis, it is conceivable that cumulative exposure to other risk factors-such as smoking, occupational exposures, and recurrent respiratory infections-might modulate this risk. Interestingly in the present study, one patient with the Pi*MS genotype and undefined etiology for bronchiectasis presented with panlobular emphysema affecting a lower lobe, despite never having smoked.

A rare genotype identified in our cohort was Pi*MI, involving the I allele, which is classified as a variant of uncertain clinical significance.^3^ This was in a young adult, never-smoker, with a serum AAT level of 141 mg/dL, who presented with bronchiectasis of indeterminate etiology, along with coexistent upper lobe emphysema. Pulmonary function was markedly impaired (FEV_1_ = 38% of predicted), and the patient also had evidence of chronic liver disease of unknown cause. This case raises the possibility that certain rare *SERPINA1* variants, although not classically associated with severe deficiency, may contribute to complex phenotypes involving both pulmonary and extrapulmonary manifestations. Notably, despite serum AAT levels within the normal range, the functional activity of the molecule may be impaired, as described for certain variants that produce structurally unstable or dysfunctional proteins.^19^ ^,^ ^20^

Another point to be considered is that chronic inflammation, a hallmark of bronchiectasis,^21^ may increase AAT concentrations due to its role as an acute-phase reactant.^10^ ^,^ ^20^ Thus, diagnosing AATD solely on the basis of the initial serum AAT level, without subsequent confirmation using complementary diagnostic tools (such as genotyping, phenotyping, or gene sequencing), may lead to underdiagnosis in this population.

Regarding microbiology findings, data from the U.S. Bronchiectasis Registry previously demonstrated increased nontuberculous mycobacterial (NTM) infections in patients with AATD.^14^ This may be explained by at least two mechanisms: AAT enhances macrophage-mediated clearance of intracellular mycobacteria through increased phagolysosomal fusion and autophagy,^22^ and it potentiates TNF-α activity. Thus, low serum AAT levels may reduce TNF-α-mediated immune responses, increasing susceptibility to mycobacterial infections.^23^ Although this pathophysiological rationale exists, no significant differences were observed in AAT variant carriers in this study.

In the present study, no other clinical, laboratory, or radiological variables differed significantly between patients with and without *SERPINA1* variants. Thus, apart from low serum AAT levels, diffuse or basal emphysema distribution, and panlobular emphysema, no consistent pattern could be identified to suggest the presence of *SERPINA1* mutations in individuals with bronchiectasis. Importantly, these findings should not be used as exclusive criteria to guide genetic investigation, since, as previously discussed, a substantial proportion of individuals carrying *SERPINA1* mutations may present with normal serum AAT levels and without radiological evidence of emphysema.

This study has limitations. This is a cross-sectional analysis based on consecutive patients evaluated in a referral center over a defined period. As such, no formal sample size calculation was performed. The single-center nature of the study limits the generalizability of the findings. Furthermore, the rarity of bronchiectasis associated with AATD led to a relatively small number of cases, potentially reducing the statistical power for detecting significant associations. Additionally, serum AAT levels were measured using turbidimetry, which, although the method available at our center, is known to be less sensitive than nephelometry-the technique preferably indicated by consensus.^3^ This lower sensitivity is particularly relevant for detecting subtle differences at lower AAT concentrations. Despite these limitations, our study provides a comprehensive clinical, microbiological, radiological, and genetic characterization of a significant population of patients with non-cystic fibrosis bronchiectasis in a referral center. Although numerous studies have explored bronchiectasis among individuals with AATD, investigations focusing on screening for AAT variants in populations with primary bronchiectasis, especially at referral centers, remain scarce. 

In conclusion, our findings suggest that AAT variants are not uncommon among patients with bronchiectasis. Clinicians should consider genotyping or further diagnostic evaluation even in patients with normal AAT levels, particularly when bronchiectasis remains unexplained. Although the identification of a *SERPINA1* mutation alone may not always define the etiology of bronchiectasis, it should be considered an additional risk factor that may act synergistically with other environmental or infectious exposures to influence disease expression. 
